# Evaluation of the Storage Stability and Quality Properties of Fresh Noodles Mixed with Plasma-Activated Water

**DOI:** 10.3390/foods11010133

**Published:** 2022-01-05

**Authors:** Yawei Wang, Yuchang Liu, Man Li, Meng Ma, Qingjie Sun

**Affiliations:** School of Food Science and Engineering, Qingdao Agricultural University, Qingdao 266109, China; wyw990708@163.com (Y.W.); 15854799863@163.com (Y.L.); mamengss@163.com (M.M.); phdsun@163.com (Q.S.)

**Keywords:** fresh noodle, plasma-activated water, storage, quality

## Abstract

Enhancing the quality retention of fresh noodles remains challenging. In this study, we investigated the effect of dough mixing with plasma-activated water (PAW) of different activation times on the storage stability and quality characteristics of fresh noodles. It was found that the total plate count in the fresh noodles prepared by PAW (PAWN) showed no obvious inhibition during storage at 25 °C, but could be significantly reduced at 4 °C as compared with the control. The decrease in L* value and pH of the PAWN was significantly retarded during storage, indicating an enhanced storage stability. The stability time of dough mixed with PAW could be significantly improved. PAW treatment decreased the viscosity properties and setback value of starch, while enhancing the interaction of water and non-water components in fresh noodles. In addition, dynamic polymerization and depolymerization of proteins were detected in Size-Exclusion High-Performance Liquid Chromatography (SE-HPLC) profiles of PAWN. The hardness and adhesiveness of the cooked noodles decreased, while the springiness significantly increased. These results implied the potential of PAW in improving the storage stability and quality of fresh noodles.

## 1. Introduction

Noodles originated in China, with a history of more than 4000 years. Noodles constitute a kind of healthy and delicious food that is easy to cook, convenient, and rich in nutrition [1]. Noodles can be served as a staple food or fast food; they have long been consumed and are increasingly popular among people worldwide [2].

Fresh noodles, as a traditional staple food, attract more consumers due to their natural flavor and better taste compared with instant fried and dry noodles [3]. The commercial value of fresh noodles is affected by their storage stability, appearance, mouthfeel, and cooking properties. However, a primary drawback of fresh noodles is their extreme vulnerability to deterioration, which was due to their high moisture content and rich nutritional substance that are favorable for microbial growth and physiological–biochemical reactions [4]. Freshly produced noodles will soon deteriorate regardless of being stored at room temperature or being refrigerated. This defect has caused huge waste in the food industry and may even result in potential food poisoning. 

A lot of effort has been made to extend the storage stability of fresh noodles. Chemical preservatives, such as calcium propionate and potassium sorbate [3]; natural preservatives, such as linseed, organic acids, and Maillard reaction products of chitosan and xylose [5]; physical sterilization, such as radiation [6] and ozone [7]; as well as modified atmosphere packaging (MAP) [8], have been demonstrated as effective technologies for the preservation of fresh noodles. However, enhancing the quality retention of fresh noodles remains challenging. Innovative preservation technologies or their combination have been continuously developed to promote the industrial production of this traditional staple food.

Plasma technology as a novel and green sterilization technology has been applied to inactivate microorganisms. It has the advantages of convenient operation and being harmless to the human body [9]. Plasma-activated water (PAW) is a kind of water disinfector prepared from distilled water treated with non-thermal plasma [10]. A variety of plasma sources are used to activate water, including direct current (DC), low frequency discharge, radio frequency discharge, and pulse corona. PAW can effectively inhibit a variety of microorganisms, such as *E. coli*, *Saccharomyces cerevisiae*, and *Staphylococcus aureus* [11]. PAW, as a bacteriostatic technology, has advantages including less adverse impacts on the environment and no possibly dangerous chemicals [12]. Traditional chemical disinfectants, particularly chlorinated products, have caused an increasing number of people to be concerned about chlorinated organic carcinogenic compounds. As a green disinfection technology, PAW is a promising alternative to traditional disinfectants in agriculture (fruit and vegetable disinfection) and the food industry (e.g., poultry product disinfection) [13].

Although the potential of plasma treatment for the preservation of many food products has been proven, its application on wheat-based food is limited. Chen et al. [14] used nonthermal plasma treatment for the sterilization of the surface of fresh wet noodles and found a decrease in the total bacterial count. Additionally, the fresh noodles showed no undesirable changes in color, texture, water distribution, and acidity. However, direct plasma treatment can only act on microorganisms on the fresh noodles’ surface. The treating capacity of plasma on the noodle surface is also limited to a single time; thus, it is difficult to use for industrial large-scale production. Moreover, studies have confirmed the potential of plasma to improve the functionality of wheat flour, such as its viscosity properties, endothermic enthalpies, and crystallinity [9]. However, few studies have investigated the effect of PAW treatment on the storage properties and quality characteristics of fresh noodles. In this study, Total Plate Count (TPC) was used to characterize the quality changes during storage; the quality of noodles was characterized by measuring the viscosity of starch and the dynamic polymerization of protein. Wheat flour was mixed with PAW to produce fresh noodles, aiming to investigate the potential of PAW for preserving fresh noodles, including inhibiting the microbial growth and darkening, as well as reducing the drop of pH value. The changes in the mixing properties of wheat dough, the cooking and textural qualities of fresh noodles, water distribution, as well as the viscosity properties of starch and the polymerization behaviors of the protein component are systematically discussed in this paper to understand the macroscopic and the structural changes of fresh noodles affected by PAW treatment. The results may provide essential data for a more complete understanding of the effect of PAW treatment on the storage properties and quality characteristics of fresh noodles.

## 2. Materials and Methods

### 2.1. Materials

Zhongyu specially refined wheat flour was supplied by Zhongyu Food Co., Ltd. (Binzhou, China), with carbohydrates, protein, and fat contents of 74.12%, 11.85%, and 0.59%, respectively. All chemicals and reagents used were of analytical grade. 

### 2.2. Preparation of Plasma-Activated Water (PAW)

**Figure 1:**
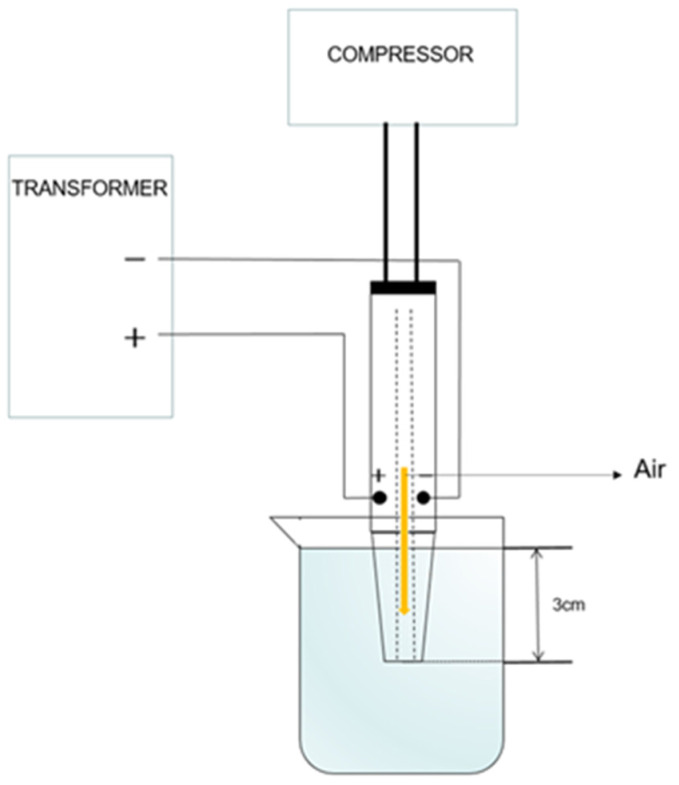
Schematic diagram of the plasma-activated water device.

Atmospheric air pressure plasma was used to produce PAW; a schematic diagram of the plasma device and the production process is shown in Figure 1. Distilled water (200 mL) was poured into a specialized beaker and treated with the atmospheric pressure plasma equipment (Model T-SPOTS25002K, TIGRES, Hamburg, Germany). The activation times were set at 0 s (control), 5 s, 10 s, 30 s, 60 s, 120 s, and 300 s, and the activated water samples were, respectively, recorded as PAW0 (control), PAW10, PAW30, PAW60, PAW120, and PAW300. The pH value and the oxidation reduction potential (ORP) of PAW were measured using a pH/ORP meter (PB-10, Sartorius Co., Gottingen, Germany).

### 2.3. Preparation of Fresh Noodles

The formula for fresh noodle preparation contains 100 g wheat flour and 34 mL sterile distilled water or PAW, which were mixed with a KitchenAid mixer (St. Joseph, MI, USA). The formed dough crumbles were placed in a sterilized sealing bag and left to rest for 20 min. Then, the noodle sheet was formed by dough crumbles passing through a small experimental noodle machine (Model JMTD-168/140, Beijing, China) and were cut into noodle strings (width, 1 mm; thickness, 0.9 mm). The noodle strings were cut to 20 cm. The noodle samples were named PAWN-0 (control), PAWN-10, PAWN-30, PAWN-60, PAWN-120, PAWN-300, according to the different amounts of PAW added. All samples were placed in sterilized aluminum foil bags and stored at 4 °C and 25 °C, respectively. To simulate the factory production environment. UV-sterilization was used in the laboratory for 30 min in advance; all equipment and appliance surfaces were wiped and disinfected with alcohol before use.

### 2.4. Determination of Total Plate Count (TPC)

The fresh noodles (25 g) were placed in 0.85% aseptic saline (225 mL) and homogenized according to the national standard GB 4789.2-2016 [15]. The homogenate was then prepared with sterile normal saline. The appropriate dilutions (1 mL) were cultured in the PCA for 48 ± 2 h at 36 ± 1 °C. The colony-forming unit (CFU) was calculated by the formula that counts the total number of bacterial colonies in the plate. TPC determination was carried out in triplicate and expressed as log_10_ CFU/g of noodle weight. To eliminate the influence of microorganisms in the environment, the determination of microorganisms was carried out in the asepsis room using a super clean table. 

### 2.5. Color and pH Measurement of Fresh Noodles

A portable Konica Minolta Chroma Meter (Model CR-400, Tokyo, Japan) was used to measure the color changes of PAWN sheets during storage using the CIE 1976 L*, a*, and b* color scale [16]. The L* value is a measurement of brightness (0–100), which can be used as an indicator of noodle darkening. The samples were cut into pieces, approximately 5 cm in diameter, and measured within 5 min of cutting and during storage.

The pH of fresh noodles was measured using the pH/ORP meter (PB-10, Sartorius Co., Gottingen, Germany). A total of 10 g of the noodle samples were ground and mixed with 100 mL distilled water.

### 2.6. Analysis of Farinographic Properties of Wheat Dough

PAW with different activation times were freshly produced and maintained at 30 °C before testing. The behavior of the PAW-mixed dough during development and mixing was tested using a farinograph (Brabender, Duisburg, Germany); the wheat flour was first weighed into the mixing bowl equipped with a thermostat, which operated at 30 ± 0.2 °C, with the reasonable quantity on the basis of its water content. The dough development time (DDT) and the stability time (DST) were measured (ICC 115/1).

### 2.7. Viscosity Analysis

The pasting properties of the sample were determined with a Rapid Visco Analyzer (RVA-TECMASTER, Perten, Australia). The noodle samples were lyophilized and ground into powder. Then, the powder (3.00 g) was mixed with deionized water (25 g) and manually homogenized with the plastic paddles 10 times to RVA testing. The tests were carried out under a heating and cooling cycle, according to American Association of Cereal Chemists (AACC) method 76-21 [17]. The pasting curves were conducted in the standard 1 method during the test. The mixed samples were held at 50 °C for 1 min, and heated from 50 °C to 95 °C at a rate of 12 °C/min, then kept at 95 °C for 2.5 min, and finally, cooled from 95 °C to 50 °C at a rate of 12 °C/min and held at 50 °C for 1.5 min. The paddle speed was at 960 rpm for the beginning 10 s, and then kept at 160 rpm until the end of the test procedure.

### 2.8. Low-Field-1H Nuclear Magnetic Resonance (LF-^1^H NMR) Analysis

LF-^1^H NMR measurements of the PAWN sheets were performed with a low-field 23-MHz NMR analyzer (NMI20-040V–I, Niumag Co., Ltd., Suzhou, China). The fresh noodle samples (5 g) were sealed with preservative film to prevent water evaporation and then put in 25-mm diameter glass tubes [18]. The transverse relaxation time (T_2_) was measured by the Carr–Purcell–Meiboom–Gill (CPMG) sequence at 32 °C. 

### 2.9. Size-Exclusion High-Performance Liquid Chromatography (SE-HPLC) Analysis

The Sodium dodecyl sulfate (SDS) solubility and molecular weight distribution profiles of proteins in PAWN were analyzed using a SE-HPLC system equipped with UV detectors (LC-20AT, Shimadzu, Japan), as described by Zhang et al. [3]. The freeze-dried powdered noodles sample (15 mg) was mixed with 1 mL of 50 mM sodium phosphate buffer with 1% SDS (phosphate buffered saline (PBS), pH 7.0). Then, the solution was vortexed for 2 h to extract SDS-soluble proteins, and the mixture was centrifuged at 8000× *g* for 10 min to collect the supernatant. Each supernatant (20 μL) was injected into a TSK G4000-SWXL column (Tosoh Biosep, Yamaguchi-shi, Japan) after filtering with a 0.45 μm Millipore filter and eluted with the PBS at a flow rate of 0.7 mL/min. The elution curve was determined at 214 nm and the column temperature was as 30 °C. The peak areas were calculated to characterize the solubility of proteins in SDS and the molecular weight distribution. 

### 2.10. Textural Properties 

Twenty-five fresh noodle strings (20 cm, 0.75 g average weight) were cooked in 450-mL boiled distilled water, until the optimal cooking time (4 min). A texture analyzer (Model TA-XT plus, Surrey, UK) was used to determine textural properties, with the following testing parameters: pre-test, test, and post-test speeds, 0.8 mm/s; compression ratio, 75%; and interval time, 2 s. In each measurement, three noodle strings were placed parallelly on the testing plate of the texture analyzer and repeated six times. The hardness, adhesiveness, springiness, and chewiness of the cooked PAWN were determined and automatically calculated by analysis software equipped with texture profile analysis (TPA). 

### 2.11. Statistical Analysis

All data analysis was performed with SPSS 20.0 Package (SPSS Inc., Chicago, IL, USA). Analysis of variance (ANOVA) was used to determine significant differences among the results (means ± standard deviation); the significant difference and mean values were obtained by Duncan’s test at a confidence level of *p* < 0.05.

## 3. Results and Discussion

### 3.1. Microbial Growth in Fresh Noodles Produced with PAW

**Figure 2:**
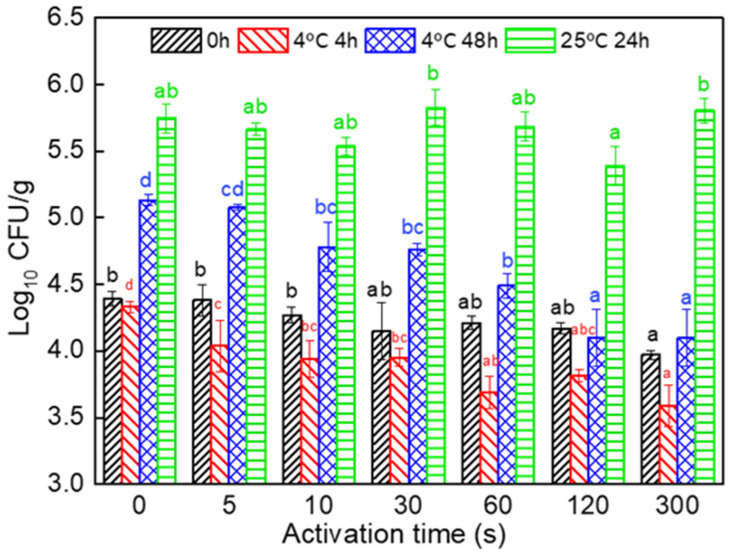
TPC changes in PAWN during storage. The different letters indicate significant differences among samples in the same storage time (*p* < 0.05).

In this study, both the control and the PAW-treated fresh noodles were made with sterile distilled water. Thus, the initial microbial quantity of freshly produced noodles could not be affected by the microorganisms in water. Figure 2 shows the changes in the TPC of the control and the PAWN with different PAW activation times during storage at ambient (25 °C) and low (4 °C) temperatures. Previous studies reported that plasma can significantly inactivate a certain number of microorganisms in water [10]. This might be attributed to the production of various reactive chemicals, such as H_2_O_2_, OH•, O_3_, nitrate, and nitrite anions, when water is activated by plasma. These reactive species can destroy the cell wall and cell membrane, which is generally agreed to make a critical difference in the inactivation of microbial cells. Additionally, the plasma-activated nitrate and nitrite in the water can help acidify the water, which will then increase the antibacterial effect of the PAW [19]. 

**Figure 3:**
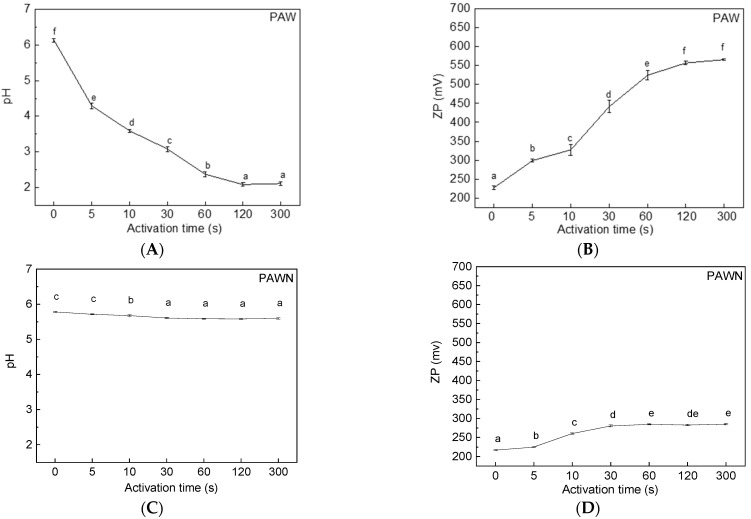
The pH (**A**) and ORP (**B**) of PAW change with the treating time, the pH (**C**) and ORP (**D**) of PAWN change with the treating time. Different letters represent significant differences (*p* < 0.05).

In this study, the initial microbial quantity in the PAW-treated fresh noodles was slightly lower than that in the control sample, while PAWN-300 presented a significant difference (Figure 2), which might be because the reactive species increased ORP and reduced pH value. As shown in Figure 3A,B, the pH of the PAW gradually decreased from 6.10 (PAW0) to 2.08 (PAW300), and the positive ORP value increased from 227.5 mV to 566.0 mV. It should be noted that the TPC decrease in the PAWN, although significant at PAW300 compared with the control, cannot acquire a potent lethal result, as reported by PAW treatment on the surface of fruits [11]. It is possible that the neutralization effect of the wheat flour matrix on the acidified PAW and the quenching effect on the reactive species were existent. As shown in Figure 3C,D, the decrease in the pH value and the increase in the ORP of the PAWN (homogenate) were much lower compared with those of PAW.

Generally, the TPC in PAWN expressed no significant decrease during storage at 25 °C for 24 h; the TPC of all samples were in the same order of magnitude with no regularity (Figure 2). However, it was quite interesting to detect that, during storage at 4 °C, the TPC in all PAWN significantly decreased, even after storage for 4 h only, compared with the freshly produced noodles (0 h), and it showed a further reduction with increasing plasma activation time. The TPC in the control (PAWN-0) was 4.33 log_10_ CFU/g after storage for 4 h at 4 °C, while those in PAWN-10 and PAWN-300 were 3.94 and 3.56 log_10_ CFU/g, respectively. After storage for 48 h at 4 °C, the microbial growth in the PAWN was also significantly inhibited, with the TPC in PAWN-120 reduced to 4.08 log_10_ CFU/g compared with 5.15 log_10_ CFU/g of the control. This might be due to the reactive chemical species generated in the PAW, which could damage or rupture the cell structure of microorganisms and might result in a “sublethally injured” or “intermediately damaged” status of microbial cells, reducing the initial microbial quantity in fresh noodles. When stored at 25 °C, the injured cells might recover due to the suitable temperature and nutrient-rich substances in fresh noodles that would be beneficial to the growth and repair process of microbial cells. Therefore, the TPC of all PAWN samples could not show inhibition after storage for 24 h at 25 °C. However, at 4 °C, the undesirable storage temperature for microbial activity could further enhance the action of PAW on the microorganisms in fresh noodles, and the self-repair of the sublethally injured cells could be delayed or inhibited. These would induce a significant decrease in the TPC immediately after 4 h and extend the microbiological storage stability of fresh noodles during low temperature storage. Zhao et al. [20] found similar results in green tea infusions treated by pulsed electric fields (PEFs). A certain percentage of microbial cells in the green tea infusion suffered sublethal damage after PEF treatment; then, the recovery of sublethal cells was detected during storage at 25 °C and 37 °C, but the repair process was inhibited at 4 °C. Therefore, in this present study, it can be concluded that the microbiological storage stability of fresh noodles produced with PAW might not be prolonged when stored at 25 °C, but can be significantly extended during low-temperature storage.

### 3.2. Effect of PAW on L* Value and pH Changes in Fresh Noodles during Storage

**Figure 4:**
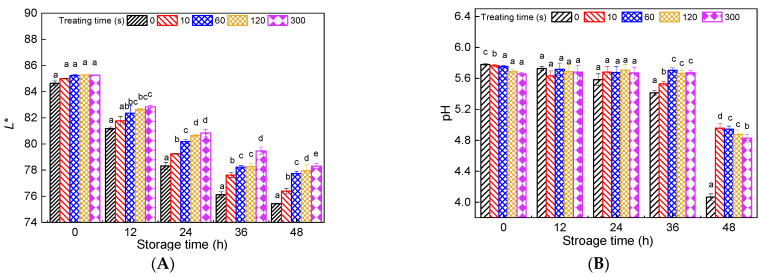
Color (**A**) and pH (**B**) change of PAWN during storage. The different letters indicate significant differences among samples in same storage time (*p* < 0.05).

The color of food is an important factor affecting sensory evaluation, which influences consumers’ evaluation of a product and whether they will buy it. For fresh noodles, a general disadvantage is that they tend to darken, which is less acceptable to consumers. To improve the storage stability of fresh noodles, it is also indispensable to stop or at least retard the darkening. The CieLab L* value measured by the Chroma meter is a function of the diffuse light reflectance (scattering) and light absorption; Li et al. [21] reported that noodle darkening can be characterized by the changes in L* value. In this study, the L* value of the freshly made PAWN slightly but not significantly increased compared with the control (Figure 4A). However, during storage at 25 °C, the darkening of the fresh noodles was significantly inhibited with the increasing PAW activation time. The L* value of the control largely decreased from 84.7 (0 h) to 75.2 after storage for 48 h, while that of PAWN-300 was 78.9 after 48 h. This was mainly due to the various reactive chemical species generated in PAW, especially the oxidative species, such as H_2_O_2_ and O_3_ [10], which could gradually oxidize the yellow pigment in fresh noodles and result in a whiter color. These active species might also prevent the polyphenol oxidase (PPO) from catalyzing phenolic compounds into colored melanin products [7]. 

For the freshly made noodles, the pH value of PAWN slightly decreased with the increase of the treating time (Figure 4B) due to the high acidity of PAW, as discussed above. During storage at 25 °C, the pH value of all samples significantly decreased with the extension of the storage time (48 h). However, the pH value of PAWN decreased much slower compared to the control during storage. These findings demonstrated that PAW might suppress the metabolic ability of microorganisms to produce acid, although showing no obvious inhibition of microbial growth. 

### 3.3. Effect of PAW on Mixing Properties of Dough

**Figure 5:**
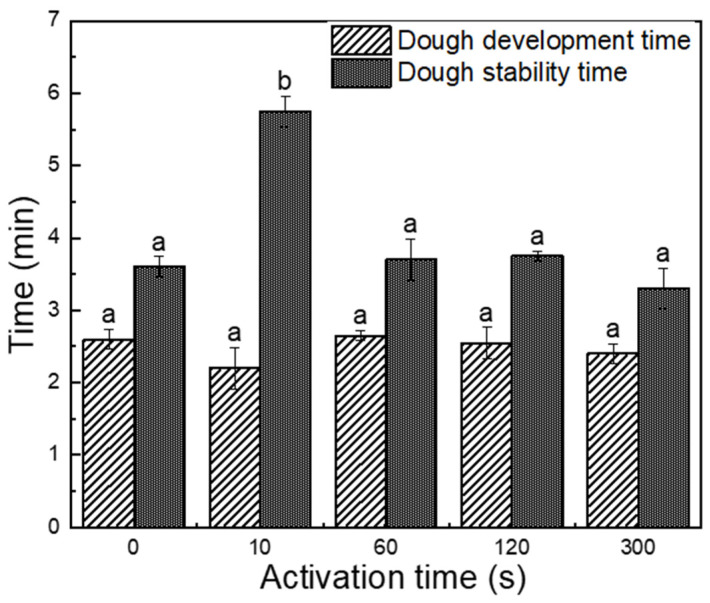
Changes in the development and stability time of wheat flour dough. The different letters indicate significant differences among samples in different activation times (*p* < 0.05).

Dough development time (DDT) is the time from the first addition of water to the development of maximum dough consistency [22]. As shown in Figure 5, PAW induced no significant difference in DDT of all dough samples. Dough stability time (DST) is described as the time difference between the point at the top of the curve where it first intersects the 500 FU line and the point at the top of the curve where it leaves the 500 FU line [23]. Generally, DST gives indications of the tolerance of the wheat flour to mix. DST increased up to 5.75 min when the PAW activation time was 10 s, and then decreased with the increasing activation time. The increase in stability time might be due to the polymerization of gluten proteins induced by the oxidative species in PAW, while excessive oxidation might be adverse to the formation of gluten network. 

### 3.4. Effect of PAW on the Changes in Viscosity Properties of Starch in Fresh Noodles

**Figure 6:**
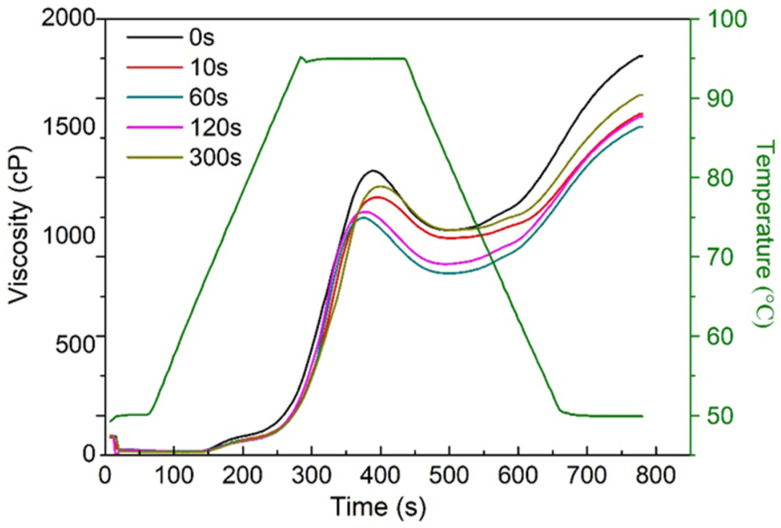
Pasting curves of PAWN samples.

The effects of PAW on the viscosity properties of the starch were evaluated (Figure 6). Many studies have shown that the viscosity properties of starch in noodles are related to their qualities, and any structural change in granular starch will be reflected in the pasting properties of starch. As presented in Figure 6, both the peak viscosity and the final viscosity of starch in fresh noodles gradually decreased with the increasing PAW activation time, from 0 s to 60 s. These changes should also be owing to the dynamic accumulation of the various reactive chemical species in PAW, which inhibited the swelling and the rupture of the starch granules. According to Dias et al. [24], the hypochlorite generated in PAW could promote a decrease in the peak viscosity of starch due to the depolymerization of amylose and amylopectin molecules in the starch granules. The accumulation of H_2_O_2_ and hypochlorite in PAW generated more oxidized starch when fully mixed with the flour during the RVA test, leading to the partial fracture of the glycosylic bonds and reducing the molecular weight of the starch molecules, which also contributed to the reduction of viscosity [25]. The rapid descent in the pH value of fresh noodles with the increasing PAW activation time, from 0 s to 60 s, might also result in the decreased viscosity of starch components. 

However, both the peak viscosity and the final viscosity increased with the further extension of the activation time from 60 s to 300 s. It might be because that the accumulation of O_3_ in PAW during the increasing activation time might increase the ability of flour to combine with water, then resulting in the enhanced viscosity [7]. It is worth mentioning that the simple sugars and smaller fragments produced due to depolymerization are easily filtered out during the cooking process and can also increase the pasting viscosity. In addition, the leaching of more starch granules was also a result of the increase in viscosity [26]. The simple sugars, such as glucose and maltose, produced by depolymerization could keep a higher water content and cause an increase in the viscosity of starch [27]. The plasma species induced starch etching, which might lead to an increase in hydrophilicity and reduce the barrier between water and starch granules. 

In addition, the setback value (final viscosity–hot paste viscosity) of the PAWN was significantly decreased compared with the control. Setback value reflects the degree of retrogradation of starch paste, and a lower setback value is beneficial to the quality and storage stability of wheat flour products [16].

### 3.5. Changes in Water Status of PAW-Treated Fresh Noodles 

**Figure 7:**
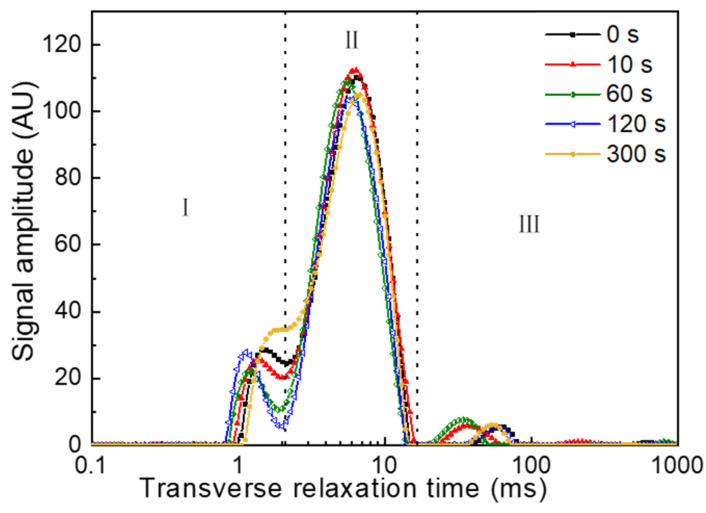
The spin–spin relaxation time (T_2_) changes of water molecules in fresh noodles samples. Area (Ι, ΙΙ, ΙΙΙ) represents bound water, weakly bound water and free water.

Water plays a vital role in the process of food preservation and storage. The fixation of water can often delay the growth of microorganisms to extend the storage stability of food [28]. In this study, the distribution of the T_2_ relaxation time of the fresh noodles was investigated to characterize the changes in the water status induced by PAW. The T_2_ relaxation time represents the diffusion and chemical exchange process between water molecules and biopolymers or other solutes [29]. Three different distribution regions (Figure 7, I–III) were detected in all noodle samples, indicating that PAW did not change the water domains in the noodle samples [30]. In general, the shorter the relaxation time, the tighter the binding between water and non-water components, showing stronger a water–solid interaction [29]. Compared with the control, the peak relaxation time of the three regions in the PAWN samples (10–120 s) slightly shifted to the left; for the PAWN-300 samples, the area covered by Region I obviously increased (Figure 7). These results demonstrated the enhanced interaction of water and non-water components of PAWN, which may also be beneficial for the storage stability of the products.

### 3.6. SDS Extractability and Molecular Weight Distribution Profiles of Proteins

SE-HPLC is a technique used to measure the content of SDS extractable proteins in wheat and their molecular weight distribution profiles, which can describe the degree of PAW-induced protein cross-linking. According to Han et al. [30], the chromatogram of SE-HPLC can be divided into four sections according to the elution time: large glutenin polymers (P1), medium glutenin polymers (P2), monomeric proteins (P3), and peptides and amino acids (P4), respectively. The proteins not soluble in SDS are defined as glutenin macropolymer (GMP).

**Figure 8:**
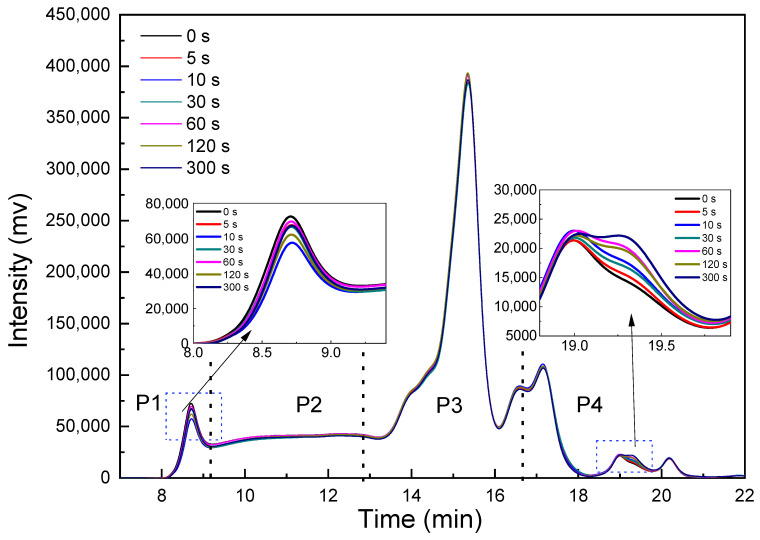
SE-HPLC chromatogram of PAWN. SE-HPLC fractions: P1 represents large glutenin polymers, P2 represents medium glutenin polymers, P3 represents monomeric proteins, P4 represents peptides and amino.

As shown in Figure 8, interestingly, the peak area of P1 decreased in PAWN-5 and PAWN-10 samples and then increased with PAW activation time for 30–300 s. A slight increase in the area of P4 was also detected. These results indicated the dynamic polymerization and depolymerization of proteins in noodle dough mixed with PAW. The reactive oxidative species in PAW could affect the structure and cross-linking of gluten proteins. This may involve the disulfide (S–S) bonds in proteins, which refer to the functional group with the structure disulfide (S–S) bonds. This is usually formed by coupling two mercaptan groups, which play a vital role in the three-dimensional structure of proteins. Appropriate oxidation would promote the formation of S–S bonds by free S-H, resulting in the polymerization of gluten proteins and the production of GMP, which could not be extracted by SDS, resulting in the significant decrease in the area of the first peak. Bahrami et al. [31] used plasma to treat wheat flour and found that the proteins presented a trend toward higher molecular-weight fractions, which indicated protein oxidation. However, the bond energy of S–S is weaker than those of C–C and C–H; excessive oxidation may further lead to S–S oxidative fracture under acidic conditions [32]. Therefore, with the extension of the treating time, the original S–S may also be partially fractured; thus, the peak area would increase again. The increase in the peak area of P4 may be due to the oxidation of the S–S bond of GMP, which would produce substances with smaller molecular weight. 

It is worth noting that the changes in the molecular weight distribution profiles of proteins were similar with the previous results of DST in Figure 5, from which it could be further concluded that the enhancement of DST might be caused by the polymerization of gluten proteins. Niu et al. [33] and Primo-Martan et al. [34] found that increased GMP content results in improved dough stability time, which indicates the higher gluten strength. However, when the PAW activation time was more than 10 s, the excessive oxidation might further lead to S–S oxidative fracture under acidic conditions, and then lead to the decline of stability time.

### 3.7. Effect of PAW on Textural Properties of Fresh Noodles

The changes in the textural properties of PAWN are summarized in Table 1. With increasing activation time, the hardness and chewiness of fresh noodles decreased. This might be due to the decrease of the setback value of starch, which was mentioned above in Figure 6. On the other hand, as reported by Baik et al. [35], the amylose content of starch was positively correlated with hardness, so this decline might also be due to the depolymerization of amylose molecules oxidated by H_2_O_2_ and hypochlorous acid [25]. The adhesiveness of cooked noodles showed a decreasing trend, which was positively correlated with the amount of amylopectin chains [36], which may also be caused by the oxidation of H_2_O_2_ and hypochlorous acid [24]. However, it was interesting that, with the increasing activation time, the springiness of fresh noodles increased, which might be due to the enhanced gluten network of fresh noodles treated by PAW. Generally, a noodle with the appropriate higher springiness is more desirable [37]. This may be because the reactive species in PAW promoted protein aggregation, and the gluten network of fresh noodles then wrapped the starch particles, leading to the increase of springiness [21]. It might also be because the hydrophily of carboxyl was enhanced by the reactive species in PAW, which affected the transfer of water by enhancing the water and non-water interactions in the noodle matrix, thus strengthening the gluten network.

## 4. Conclusions 

PAW was concluded as a potential technology to enhance the storage stability of fresh noodles without affecting the eating quality. Mixing with PAW significantly reduced the initial TPC content and further inhibited microbial growth in fresh noodles during storage. The decrease in the L* value and pH of fresh noodles was also retarded.

PAW decreased the viscosity properties and significantly inhibited the retrogradation of starch, which might contribute to the decreased hardness of cooked noodles. Additionally, PAW treatment induced the dynamic polymerization and depolymerization of gluten proteins, and this might result in the increase in enhanced dough stability and springiness of noodles. Further studies should be performed on the combination of PAW and other preservation technologies to further prolong the shelf life of fresh noodles.

## Figures and Tables

**Table 1:** The influence of different PAW on the texture properties of samples.

Quality Parameters	Activation Time (s)				
	0	10	60	120	300
Hardness (g)	6750.89 ± 241.99 ^b^	5757.49 ± 61.98 ^a^	5624.21 ± 171.55 ^a^	5310.20 ± 101.74 ^ba^	5351.96 ± 185.47 ^a^
Chewiness (g)	3608.09 ± 188.96 ^c^	3025.74 ± 178.57 ^ab^	3059.79 ± 64.33 ^b^	2791.09 ± 310.56 ^ab^	2726.69 ± 185.59 ^a^
Adhesiveness (g·s)	450.80 ± 71.27 ^b^	232.82 ± 68.95 ^a^	215.25 ± 24.95 ^a^	186.10 ± 27.43 ^a^	190.29 ± 25.61 ^a^
Springiness	0.85 ± 0.05 ^a^	0.90 ± 0.05 ^a^	0.93 ± 0.02 ^b^	0.92 ± 0.03 ^b^	0.92 ± 0.03 ^b^

Means with different small letter superscripts within the same rows are significantly different at *p* < 0.05.

## Data Availability

Not applicable.
